# Evaluation of MRI-Based Measurements for Patellar Dislocation: Reliability and Reproducibility

**DOI:** 10.3390/diagnostics15202647

**Published:** 2025-10-20

**Authors:** Ivan Brumini, Tamara Pranjkovic, Danijela Veljkovic Vujaklija

**Affiliations:** 1 Department of Diagnostic and Interventional Radiology, Clinical Hospital Center Rijeka, 51000 Rijeka, Croatia; brumini92@gmail.com; 2 Faculty of Health Studies, University of Rijeka, 51000 Rijeka, Croatia; 3 Medical Faculty, University of Rijeka, 51000 Rijeka, Croatia; 4 Pula General Hospital, 52100 Pula, Croatia; tamara.pranjkovic@gmail.com

**Keywords:** knee, MRI, patellar dislocation, prognosis

## Abstract

**Background/Objectives:** The aim of our study was to identify the most reliable MRI measurements associated with patellar dislocation. **Methods:** MRI scans from 86 knees (48 controls and 38 with a history of patellar dislocation) were retrospectively analyzed. The following parameters were measured: lateral trochlear inclination (LTI) and its modified version, sulcus angle (SA), trochlear depth (TD), tibial tubercle–trochlear groove distance (TT–TG), patellar tendon–lateral trochlear ridge distance (PT–LTR), and PT–LTR horizontal, a novel modification. Inter-rater reliability was assessed using intraclass correlation coefficients (ICCs), and diagnostic accuracy was evaluated using ROC analysis. **Results:** All measurements significantly differed between the groups (*p* < 0.05). SA and TD were highly discriminative (AUC > 0.8) but demonstrated lower inter-rater agreement. PT-LTR horizontal strongly correlated with PT-LTR and was equally sensitive and specific for patellar dislocation as PT-LTR (81.6% and 87.5%, respectively) when in line or extending more laterally than the lateral trochlear ridge (AUC = 0.896, *p* < 0.001). LTI demonstrated the highest diagnostic performance with a sensitivity of 89.5% and a specificity of 97.9% for a cut-off ≤12.85° (AUC = 0.981), with excellent inter-rater agreement. LTI modified also performed well (AUC = 0.937), with a sensitivity and specificity of 81.6% and 93.7%, respectively. **Conclusions:** LTI, PT–LTR, and their modified versions demonstrated the highest reliability and diagnostic performance among the MRI measurements evaluated. Given their reproducibility and ease of application, these parameters may be useful in the imaging assessment of patellar dislocation. Further prospective studies are recommended to confirm their clinical utility in broader populations.

## 1. Introduction

Patellofemoral instability is a clinical condition characterized by an increased likelihood of the patella to dislocate laterally [1,2,3,4]. The prevalence of acute patellar dislocation is rather high, ranging from 43 cases per 100,000 cases among children and adolescents up to 6–77 cases per 100,000 persons per year in adults. If not treated, there is a high rate of associated injuries, particularly recurrent dislocations, and osteoarthritis [1,3].

The main predisposing factors associated with patellar dislocation are those that indicate inadequate soft tissue or bony restraints, such as trochlear dysplasia and patella alta, and those that increase the degree of laterally directed force to the patella, such as increased tibial tuberosity–trochlear groove distance (TT-TG) or tibial tubercule–posterior cruciate ligament distance (TT-PCL) [3,5,6,7].

The most significant predisposing factor for patellar dislocation is trochlear dysplasia [5,6,8]. To date, Dejour’s classification has been most widely used to assess the severity of trochlear dysplasia [6]. A plethora of quantitative measurements to characterize trochlear dysplasia has been described using plain X-ray, CT, or MRI [5,6,8,9]. Unlike other imaging modalities, MRI has multiple advantages, enabling the simultaneous evaluation of bone and soft tissue structures, including the articular cartilage, with no ionizing radiation exposure [10,11,12,13,14].

Standard measurements, such as TT-TG, lateral trochlear inclination (LTI), and the sulcus angle (SA), do not account for the containment of the patella by the trochlea [8]. Recently, patellar tendon-lateral trochlear ridge distance (PT-LTR) was described as highly reliable and discriminative for patellar dislocations, taking into account trochlear morphology and extensor mechanism lateralization in a single measurement [3].

To the best of our knowledge, no studies have yet compared the reproducibility and interobserver agreement in correlation to the patellar dislocation between these MRI measurements: LTI, LTI modified, SA, trochlear depth (TD), TT-TG, TT-PCL, lateral trochlear ridge–trochlear groove distance (LTR-TG), PT-LTR, and PT-LTR horizontal.

The aim of this study was to identify the most reliable and discriminative MRI measurements for the assessment of patellar dislocation and to evaluate the reproducibility and diagnostic performance of PT-LTR and its modification.

We hypothesized that specific MRI measurements, lateral trochlear inclination (LTI), patellar tendon–lateral trochlear ridge distance (PT-LTR), and their modified versions would be reliable and reproducible parameters for distinguishing knees with and without a history of patellar dislocation.

## 2. Materials and Methods

This study was conducted at our tertiary-care Clinical Hospital Centre. After obtaining approval from the institutional review board, we retrospectively reviewed MRI knee scans performed in our institution during a five-year period.

### 2.1. Participants

Our study group comprised 86 knee MRI scans of both male and female patients, aged 9 to 47 years. The control group comprised 48 normal or near-normal knee MRI scans referred to our institution. Control studies received MRI mainly for indications such as knee pain, swelling, and a history of trauma and had no significant radiological findings overall. Pediatric patients were included in this study because patellar dislocation commonly occurs in children and adolescents, and an early identification of anatomical risk factors is clinically important to guide management and prevent recurrence [1,2,3,4]. While it is true that skeletally immature knees may have incomplete bony morphology, MRI allows the simultaneous evaluation of both bony and soft tissue structures, including cartilage, which provides sufficient landmarks for the accurate measurement of the patellofemoral joint [9,10,11,12,13,14].

MRI knee scans of 38 patients with a history of patellar dislocation were included in the dislocation study group.

Initially, the number of MRI scans was much higher, but due to the changes in joint morphology that could result in a selection bias, patients with significant osteoarthritic change or traumatic change of the knee and patients with prior knee surgery (medial patellofemoral ligament reconstruction, tibial tubercle osteotomy, meniscal repair, and others) were excluded from both study groups. A significant osteoarthritic change was defined as at least one of the following findings: formed osteophytes and significant chondromalacia patellae with a minimum of grade II chondromalacia according to the International Cartilage Repair Society (ICRS) classification [15]. A significant traumatic change in the knee was defined as having an evident bony or osteochondral fracture or an MRI finding of moderate-to-high-grade ligamentous injury. None of the MRI scans in our study were performed in the acute post-dislocation setting.

### 2.2. MRI Scanning

All knee MRI scans were performed using a 1.5 T MR scanner and a slice thickness of 3 mm. Patients were positioned supine, with the knee placed in a dedicated coil in a neutral position with slight semiflexion (approximately 15 degrees). The examinations were performed using an adapted knee MRI protocol recommended by the European Society of Musculoskeletal Radiology (ESSR). The list of the sequences and their parameters is shown in Table 1.

**Figure 1:**
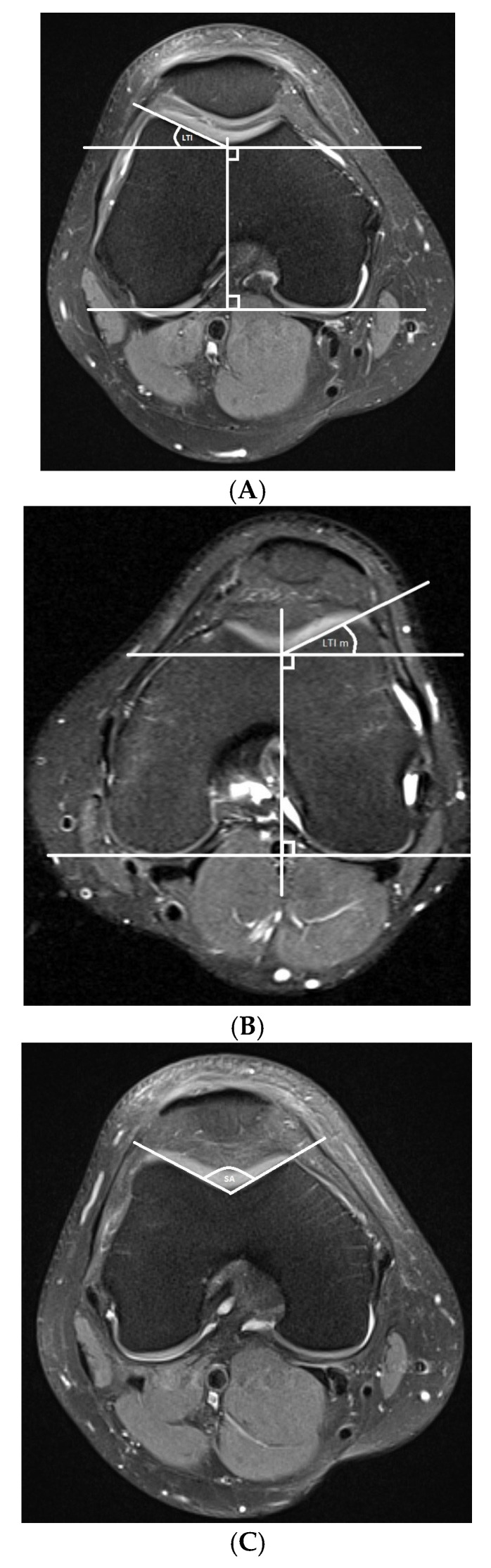

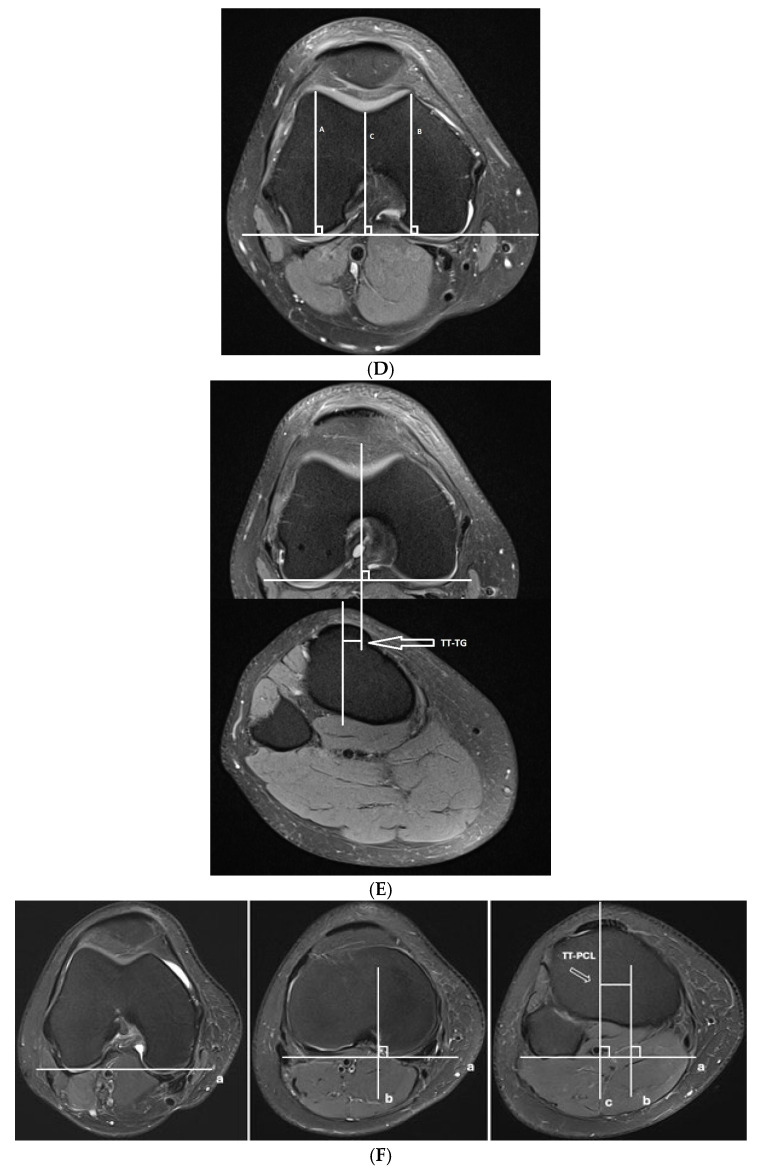
MRI measurements for the assessment of patellar dislocation. (**A**) Lateral trochlear inclination (LTI), measured between the bony contours of the lateral trochlear facet on the most proximal axial slice containing trochlear cartilage and a posterior condylar tangential line at the same level. (**B**) Lateral trochlear inclination modified (LTI m), measured between the bony contours of the lateral trochlear facet at the level of the trochlear groove and a posterior condylar tangential line at the same level. (**C**) Sulcus angle (SA), measured between the medial and lateral facets using bony landmarks for measurement purposes. (**D**) Trochlear depth (TD), assessed by measuring the maximal anteroposterior distance of the medial femoral condyle (distance A), the maximal anteroposterior distance of the lateral femoral condyle (distance B), and the minimal anteroposterior distance between the deepest point of the trochlear groove and the line paralleling the posterior outlines of the femoral condyles (distance C), all measured on the axial scan about 3 cm above the joint line. TD was calculated according to the following formula: ([A + B]/2) − C. (**E**) Tibial tubercle–trochlear groove distance (TT-TG): the maximum distance taken from the lines drawn perpendicular to the deepest area of the trochlear groove and the center of the patellar tendon insertion on the tibial tuberosity on axial images. (**F**) Tibial tuberosity–posterior cruciate ligament distance (TT-PCL): the maximum distance taken from the lines drawn perpendicular to the medial border of the posterior cruciate ligament at its tibial insertion (b) and the center of the patellar tendon insertion on the tibial tuberosity on axial images (c), parallel to the posterior condylar tangential line (a).

**Figure 2:**
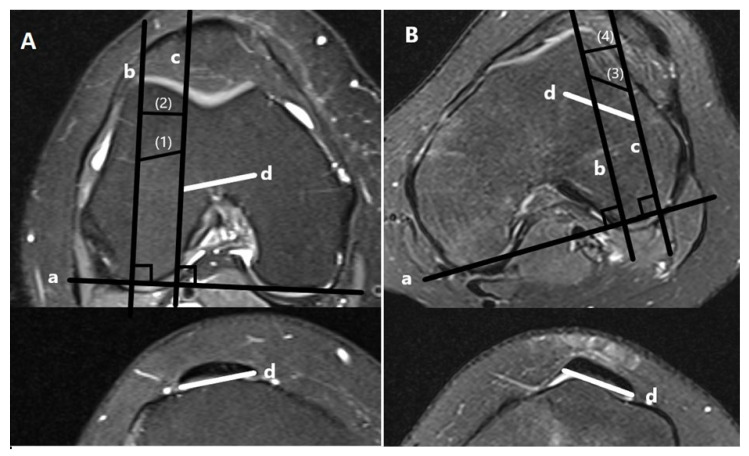
Measurement of PT-LTR oblique vs. PT-LTR horizontal on axial images from a patient without patellar dislocation (**A**) and a patient with patellar dislocation (**B**). (a) Posterior condylar axis, (b) line through the apex of the lateral trochlear ridge perpendicular to the posterior condylar axis, (c) line through the most lateral point of the patellar tendon perpendicular to the posterior condylar axis, (d) translated patellar tendon from the first distal axial scan where the patellar tendon without bone is identified. (1) PT-LTR oblique and (2) PT-LTR horizontal in a patient without patellar dislocation and (3) PT-LTR oblique and (4) PT-LTR horizontal in a patient with patellar dislocation.

For both groups, the following measurements were assessed and are shown in Figure 1 and Figure 2:

1. Trochlear dysplasia measurements
  - Lateral trochlear inclination (LTI) and a modified LTI measurement, measured at the level of the trochlear groove and named LTI modified [9,16]
  - Sulcus angle (SA) [17]
  - Trochlear depth (TD) [18]
2. Lateralization measurements
  - Tibial tubercle–trochlear groove distance (TT-TG) [6,19]
  - Tibial tuberosity–posterior cruciate ligament distance (TT-PCL) [7]
  - Lateral trochlear ridge–tibial groove distance (LTR-TG) [3]
  - The novel lateralization measurement of the patellar tendon beyond the lateral trochlear ridge distance (PT-LTR) [3]

To ensure the reproducibility of the novel PT-LTR, we measured the PT-LTR distance on the same axial MRI scans in two ways. First, we measured the width of the patellar tendon extending laterally to the lateral trochlear ridge and named it PT-LTR oblique [3]. Additionally, we measured the length of the lateral extension of the patellar tendon beyond the lateral trochlear ridge, parallel to the posterior intercondylar line, thus measuring the net lateralization of the tendon and not the width of the tendon. This measurement was named PT-LTR horizontal. For both ways of measurement, distances lateral to the trochlear ridge were allocated a positive value, and distances medial to the trochlear ridge were assigned a negative value. Distances in line with the trochlear ridge were assigned a value of zero (Figure 2).

Additionally, the axial width of the patellar tendon (WPT) was measured and used to define the center of the patellar tendon.

Two raters with advanced knowledge in musculoskeletal radiology blindly evaluated the studies.

### 2.3. Statistical Analysis

Statistical analysis was performed by using Statistica for Windows, release 13.1 (Stasoft, INC., Tulsa, OK, USA) and MedCalc (MedCalc Inc., Mariakerke, Belgium).

We evaluated inter-rater agreement using interclass correlation coefficients (ICCs) according to Koo TK and Li MY (less than 0.5—poor; between 0.5 and 0.75—moderate; between 0.75 and 0.9—good; and greater than 0.9—excellent) [20]. The correct ICC form for inter-rater reliability, in our investigation, included a two-way random-effects model and absolute agreement.

The normal distribution of continuous variables was tested using the Kolmogorov–Smirnov test, and the data of all examined variables are presented as the mean ± standard deviation (SD). Although some of these data were not normally distributed, we used the mean ± standard deviation (SD) for a simpler understanding and comparison to other papers. To test the differences between the groups according to the examined variables, we used a *t*-test for independent groups or the Mann–Whitney test, depending on the normality of distribution. The data for gender are presented as frequencies or proportions, and the analysis of the difference was performed using Pearson’s χ^2^ test.

The accuracy of a test to discriminate dislocation cases from normal cases was evaluated using Receiver Operating Characteristic (ROC) analysis. The correlation analyses were expressed by the Spearman or Pearson correlation coefficient. A comparison of ROC curves was used to test the statistical significance of the difference between the areas under different dependent ROC curves (derived from the same cases).

All statistical values were considered significant at *p* < 0.05.

## 3. Results

With respect to gender, no significant difference was determined between the control and dislocation group (*p* > 0.05). In the dislocation group, there was a female predominance (female/male; 24/14). The means and standard deviations for the age distribution of participants are given, namely, 24 ± 11 years for the control group and 23 ± 10 years for the dislocation group. The trochlear dysplasia measurements, namely, SA and TD, had poor inter-rater agreement in both groups, with the intraclass correlation coefficients being lower in the dislocation group (Table 2).

A predictability screen was performed to assess for statistically significant differences in measurements between the dislocation group and the control group (Table 3).

All of the measurements investigated demonstrated significant differences (all *p* < 0.05) for patients in the control group compared to those who sustained a patellar dislocation, except LTR-TG (*p* > 0.05) (Table 3).

A comparison of the AUC values showed that the AUC of TT-PCL and LTR-TG were significantly smaller than the others (all *p* < 0.001); so, we excluded these measurements from further analysis. Thus, sensitivity, specificity, and cut-off values were calculated for the six remaining variables.

There were strong correlations between PT-LTR and PT-LTR horizontal in both groups (control group: r = 0.989, *p* < 0.001; dislocation group: r = 0.992, *p* < 0.001) (Table 4).

**Figure 3:**
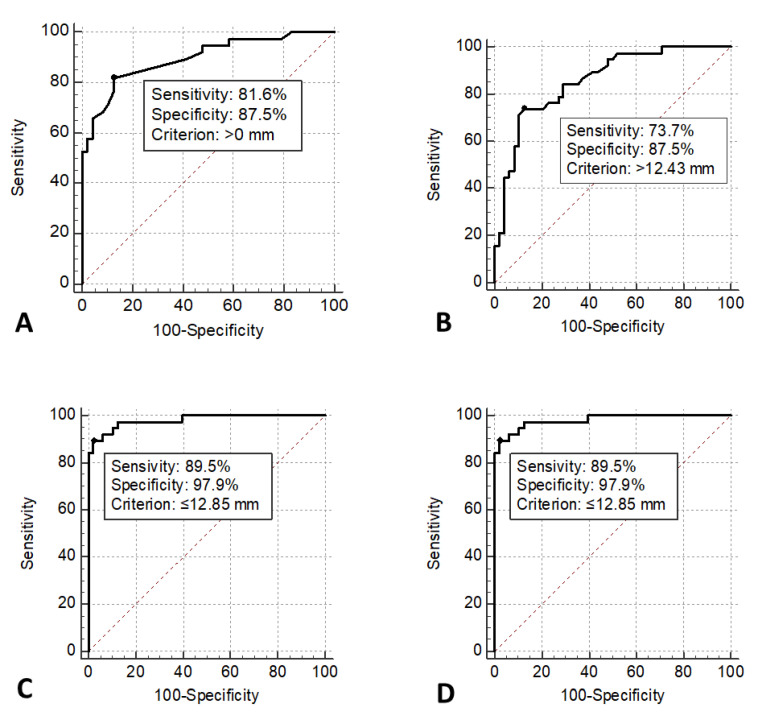
AUC of (**A**) PT-LTR horizontal, (**B**) TT-TG, (**C**) LTI, and (**D**) LTI modified, estimated by Receiver Operating Characteristic (ROC) analysis.

At the cut-off point higher than 0 (i.e., extending more laterally to the lateral trochlear ridge) for PT-LTR horizontal, sensitivity was 81.6%, while specificity was 87.5%, and at the cut-off point higher than 12.43 mm for TT-TG, sensitivity was 73.7% and specificity was 87.5% (Figure 3).

## 4. Discussion

Accounting for up to 3% of total knee injuries and with the rate of recurrent dislocations ranging from 13% to 40%, lateral patellar dislocations, if left untreated, can result in long-term disability [2,21,22,23]. Although the risk of patellar dislocation has been associated with multiple anatomical predisposing factors, including ligamentous laxity, lateralization of the tibial tubercle, trochlear dysplasia, increased TT-TG distance, and torsional deformity, only the skeletal immaturity, an SA greater than 154°, and an Insall–Salvati ratio greater than 1.3 were identified as independent predictors of redislocations [24,25]. Dejour et al. [6] found that 96% of patients with a history of a true patellar dislocation had evidence of trochlear dysplasia. This study focused on trochlear dysplasia and lateralization measurements, with other relevant factors such as patella alta measurements, patellar tilt, and patellar morphology being beyond the scope of this analysis.

While CT provides excellent spatial resolution for bony details, MRI offers the advantage of simultaneously evaluating bone, cartilage, and soft tissue structures without ionizing radiation, making it the preferred modality for comprehensive patellofemoral assessment [5,6,8,9,10,11,12,13,14].

In this study, we first investigated the MRI measurements that characterize trochlear dysplasia, which were easily measured on routine MRI scans, and then, we examined the measures of lateralization, i.e., those increasing the degree of laterally directed force to the patella [3,14].

The investigated trochlear dysplasia measures (LTI, TD, and SA) were highly discriminative for patellar dislocation, which is consistent with other studies [1,10,26,27,28]. Our results demonstrate that the mean value of LTI in knees with trochlear dysplasia that had sustained patellar dislocation is 8.33 ± 4.4 compared to 21.20 ± 3.93 in normal knees and is highly discriminative for patellar dislocation at the threshold of 12.85°, with a sensitivity of 89.5% and a specificity of 97.9%, which is similar to other studies [10,27,28]. The modification of the standard LTI measurement, i.e., LTI modified, was also highly discriminative for patellar dislocation at the threshold of 19.43°, with a sensitivity of 81.6% and a specificity of 93.7%, additionally proving the usefulness of this measurement in MRI studies, even when measured at the level of the trochlear groove, with the caveat of moderate inter-rater agreement.

SA had 89.5% sensitivity and 93.7% specificity at the threshold of 137.82°, which is similar to previous findings [18,29]. Along with LTI, TD measurements have previously been found to be helpful in distinguishing between low-grade and high-grade dysplasia [26]. Namely, our results demonstrate that compared to SA, TD measurements representing the depth of the bony trochlea are more sensitive and specific for patellar dislocation (97.4% and 85.4%, respectively) at a cut-off depth of 5.09 mm and are more sensitive, but less specific, than LTI. To avoid the variability related to the cartilage thickness, we used bony landmarks for measurement purposes, as it was previously reported that there is a statistically significant difference between the bony and cartilaginous sulcus angle [17,30].

However, in our study, both SA and TD had poor correlation between raters in both study groups, suggesting difficulties in the execution of those measurements for high-grade trochlear dysplasia, which is consistent with other studies [5,26,30,31]. On the other hand, in our study, LTI demonstrated excellent inter-rater agreement in the dislocation group. Furthermore, it is noteworthy that all the other investigated measurements that presented with better inter-rater agreement (Table 1) also presented with relatively lower intraclass coefficients in the dislocation group, confirming difficulties when analyzing dysplastic knees. Our results confirm previous findings that measures of trochlear dysplasia (SA and TD) are highly sensitive and specific, but due to poor inter-rater agreement, they are the most unreliable among the investigated measurements.

Among the measures discernible of the increased degree of laterally directed force to the patella we investigated the TT-TG, TT-PCL, LTR-TG and the PT-LTR. The TT-TG distance is a standard measurement for the evaluation of trochlear dysplasia and is often considered to indicate tibial tuberosity osteotomy and re-fixation [3,17]. MRI has been found to be reliable for assessing TT-TG [14,32]. In our study groups, the mean MRI value of TT-TG in normal knees was 9.14 ± 3.77 mm, compared to 15.09 ± 4.07 mm in dysplastic knees, and at a cut-off value greater than zero, patellar dislocations had a positive likelihood ratio of 5.89 (95% CI: 0.2–0.5). This is in accordance with findings reported in other studies [3,29,33]. Pace et al. have shown that an increased TT-TG value correlates with LTI (with a lower LTI being representative of more dysplasia) and that higher degrees of dysplasia increase the distance between the trochlear groove and the tibial tubercle [34]. It is noteworthy that there may be other underlying factors that increase the TT-TG distance, such as femoral neck anteversion, external tibial torsion, and subtalar pronation [17]. A study by Carlson et al. found that static TT-TG distance is not a strong indicator of the dynamic lateral position of the patella and cannot accurately predict dynamic lateral displacement of the patella [35]. Furthermore, in patients with severe trochlear dysplasia with convex or flat morphology, the measurement of the TT-TG distance is often difficult to measure and thus unreliable, as the deepest point of the trochlear groove cannot be accurately determined [36,37]. Hence, the practical value of the TT-TG distance may be limited in such patients. 

As already emphasized, lateralization of the tibial tubercle is a significant risk factor for the recurrence of patellar dislocation [1,6,38,39,40]. The importance of lateral forces acting on the patella has been highlighted in multiple biomechanical studies [41,42,43]. The TT-PCL was initially widely used to assess lateralization; however, this measurement focuses purely on the lateral positioning of the tibial tubercle itself, and it has rather low inter-rater agreement Hence, it should be used only in conjunction with TT-TG [44,45,46]. In our study, the TT-PCL distance was discriminative for patellar dislocation with moderate inter-rater agreement. However, TT-PCL demonstrated significantly lower sensitivity and specificity than the rest of the measurements. For this reason, and in light of the recent results by Carlson et al. [35], we further investigated the standard values and significance of PT-LTR, a novel measurement of lateralization, in our study population.

PT-LTR distance was described as highly discriminative for patellar dislocation, simultaneously reflecting extensor mechanism lateralization and trochlear morphology; yet, there are not many published studies that support this finding. In our study, PT-LTR distance was reliable and highly discriminative for patellar dislocation. Additionally, we introduced a modification of the measurement (PT-LTR horizontal) by measuring the length of the lateral extension of the patellar tendon beyond the lateral trochlear ridge, hence determining the net lateralization of the tendon reflecting the lateral force vector.

Since both variations of PT-LTR were strongly correlated, we further investigated only the newly defined PT-LTR horizontal (control group: r = 0.989; *p* < 0.001, dislocation group: r = 0.992; *p* < 0.001). At a cut-off value > 0 mm (i.e., extending laterally to the lateral trochlear ridge), PT-LTR horizontal demonstrated a sensitivity of 81.6% and a specificity of 87.5% for patellar dislocation, with a positive likelihood ratio of 6.53 (95% CI: 0.1–0.4). PT-LTR horizontal was equally specific and more sensitive for patellar dislocation than TT-TG, with a higher likelihood ratio for patellar dislocation, which could justify its clinical implementation. In our study, PT-LTR, via both ways of measurement, had moderate agreement between raters, which is not contrary to Mistovich et al., who reported even higher intraclass correlation coefficients for PT-LTR distance [3]. Moreover, a strong correlation between the two ways of measuring PT-LTR further supports the advantages of using PT-LTR as a discriminative measurement of patellar dislocation, which is easily applicable and reproducible in both ways of measurement. Nevertheless, further studies are needed to assess the eventual differences in highly dysplastic knees, especially types C and D, where the lateral trochlear ridge cannot be accurately defined.

This retrospective, single-center study was limited by a relatively small sample size due to strict exclusion criteria, which restrict the generalizability of the results. Nevertheless, the findings demonstrate statistically significant associations between MRI measurements and patellar dislocation and provide valuable initial pilot data that should be further investigated in larger, prospective cohorts. Recurrent dislocations or instability after operative management were not captured. Clinical correlation with dynamic examinations was not performed, which may have strengthened the link between MRI measurements and functional patellofemoral instability. The control group consisted of patients with minor knee complaints but no structural abnormalities, which may introduce heterogeneity; ideally, an age- and sex-matched asymptomatic control group would be preferable, although not feasible for ethical reasons. The inclusion of skeletally immature patients may have affected measurements due to incomplete ossification; however, this aligns with the epidemiology of patellar dislocation, which predominantly affects children and adolescents, and MRI provides sufficient anatomical detail for a reliable assessment. Additionally, parameters such as the Insall–Salvati Index, Caton–Deschamps Index, and patellar morphology were not analyzed, as the primary focus of this study was on trochlear dysplasia and lateralization measurements (LTI, PT–LTR, and their modifications). Finally, genders were analyzed together, despite well-documented skeletal differences across sexes and demographic groups [47], which may limit the interpretation of gender-specific findings.

## 5. Conclusions

LTI, PT–LTR, and their modified versions demonstrated the highest reliability and diagnostic performance among the MRI measurements evaluated. These parameters demonstrated good diagnostic performance and reproducibility in our retrospective cohort and could have potential value in the imaging assessment of patellar dislocation. However, further prospective studies in larger populations are needed to validate these findings and to clarify their role in clinical decision-making.

## Figures and Tables

**Table 1:** Knee MRI protocol—list of the sequences and their parameters.

	FOV (cm)	Slice Thickness (mm)	TR	TE	Matrix (At Least)
Coronal T1 SE	32	3	500	15	288 × 288
Coronal PD FS	38	3	>2500	20–30	288 × 288
Sagittal PD FS	38	3	>2500	20–30	288 × 288
Axial PD FS	38	3	>2500	20–30	256 × 256
Sagittal PD	32	0.6	1000	20–30	288 × 288

FOV, field of view; PD FS, proton density fat suppression; SE, spin echo; TE, echo time; TR, repetition time.

**Table 2:** Intraclass correlation coefficients (ICCs) among observers (inter-rater reliability) of investigated measurements for the evaluation of patellofemoral instability (PFI).

Measurement	* ICC (95% CI)	
	Control Group	Dislocation Group
PT-LTR	0.708 (0.513–0.830)	0.629 (0.221–0.821)
PT-LTR horizontal	0.729 (0.550–0.841)	0.613 (0.235–0.806)
SA	0.432 (−0.033–0.707)	0.453 (0.150–0.676)
TT-TG	0.862 (0.593–0.941)	0.788 (0.030–0.933)
LTR-TG	0.638 (0.343–0.801)	0.571 (0.121–0.793)
WPT	0.949 (0.911–0.971)	0.857 (0.735–0.924)
TT-PCL	0.671 (0.482–0.800)	0.530 (0.258–0.725)
LTI	0.871 (0.726–0.934)	0.916 (0.600–0.970)
LTI modified	0.715 (0.543–0.829)	0.542 (0.242–0.741)
TD	0.471 (−0.061–0.753)	0.378 (0.067–0.621)

* ICC less than 0.5—poor; between 0.5 and 0.75—moderate; between 0.75 and 0.9—good; and greater than 0.9—excellent inter-rater agreement.

**Table 3:** Differences in the investigated measurements between the control and dislocation group.

	Mean ± SD					
Measurement	Control Group	Dislocation Group	*p*_1_	^c^ AUC	^d^ 95% CI	*p*_2_
PT-LTR/mm	−1.90 ± 2.95	4.88 ± 4.13	<0.001 ^a^	**0.898**	0.814 to 0.953	<0.001
PT-LTR horizontal/mm	−1.79 ± 2.74	4.40 ± 3.79	<0.001 ^a^	**0.896**	0.812 to 0.952	<0.001
SA/°	130.89 ± 5.80	146.62 ± 7.79	<0.001 ^b^	**0.950**	0.880 to 0.985	<0.001
TT-TG/mm	9.14 ± 3.77	15.09 ± 4.07	<0.001 ^b^	**0.857**	0.765 to 0.923	<0.001
LTR-TG/mm	22.10 ± 3.45	22.99 ± 2.79	0.203 ^b^	0.590	0.479 to 0.695	0.146
TT-PCL/mm	19.49 ± 3.82	21.89 ± 4.97	0.013 ^b^	**0.649**	0.539 to 0.749	0.014
LTI/°	21.20 ± 3.93	8.33 ± 4.40	<0.001 ^b^	**0.981**	0.957 to 1.00	<0.001
LTI modified/°	24.58 ± 4.46	16.22 ± 3.78	<0.001 ^b^	**0.937**	0.863 to 0.978	<0.001
TD/mm	6.82 ± 1.34	3.57 ± 1.16	<0.001 ^b^	**0.972**	0.912 to 0.996	<0.001

^a^ Mann–Whitney test; ^b^ *t*-test for independent samples; ^c^ AUC, area under the curve; AUCs > 0.6 are considered discriminative (in bold); ^d^ 95% CI, confidence interval.

**Table 4:** Cut-off values, sensitivity, and specificity of the investigated measurements for the evaluation of patellofemoral instability (PFI).

Measurement	Cut-Off	Sensitivity (%)	Specificity (%)
PT-LTR horizontal/mm	>0 *	81.6	87.5
PT-LTR/mm	>0 *	81.6	87.5
SA/°	>137.82	89.5	93.7
TT-TG/mm	>12.43	73.7	87.5
LTI/°	≤12.85	89.5	97.9
LTI modified/°	≤19.43	81.6	93.7
TD/mm	≤5.09	97.4	85.4

* 0 = in line with the lateral trochlear ridge.

## Data Availability

The data supporting this study are available from the authors upon reasonable request, subject to institutional and ethical restrictions.
